# Initial in-hospital heart rate is associated with three-month functional outcomes after acute ischemic stroke

**DOI:** 10.1186/s12883-021-02252-2

**Published:** 2021-06-11

**Authors:** Ya-Wen Kuo, Meng Lee, Yen-Chu Huang, Jiann-Der Lee

**Affiliations:** 1 Department of Nursing, Chang Gung University of Science and Technology, Chiayi Campus, Taiwan; 2 Department of Neurology, Chang Gung Memorial Hospital, Chiayi, Taiwan; 3 College of Medicine, Chang Gung University, Taoyuan, Taiwan

**Keywords:** Acute ischemic stroke, Heart rate, Functional outcome

## Abstract

**Background:**

Increased heart rate (HR) has been associated with stroke risk and outcomes.

**Material and methods:**

We analyzed 1,420 patients from a hospital-based stroke registry with acute ischemic stroke (AIS). Mean initial in-hospital HR and the coefficient of variation of HR (HR-CV) were derived from the values recorded during the first 3 days of hospitalization. The study outcome was the 3-month functional outcome. Odds ratios (ORs) with 95% confidence intervals (CIs) were estimated using multivariable logistic regression analysis.

**Results:**

A higher mean HR level was significantly and continuously associated with a higher probability of unfavorable functional outcomes. Compared with the reference group (mean HR < 70 beats per minute), the multivariate-adjusted OR for an unfavorable outcome was 1.81 (95% CI, 1.25–2.61) for a mean HR ≥ 70 and < 80 beats per minute, 2.52 (95% CI, 1.66 − 3.52) for a mean HR ≥ 80 and < 90 beats per minute, and 3.88 (95% CI, 2.20–6.85) for mean HR ≥ 90 beats per minute. For stroke patients with a history of hypertension, the multivariate-adjusted OR for patients with a HR-CV ≥ 0.12 (versus patients with a HR-CV < 0.08 as a reference) was 1.73 (95% CI, 1.11–2.70) for an unfavorable outcome.

**Conclusions:**

Our results indicated that a high initial in-hospital HR was significantly associated with unfavorable 3-month functional outcomes in patients with AIS. In addition, stroke patients with a HR-CV ≥ 0.12 also had unfavorable outcomes compared with those with a HR-CV < 0.08 if they had a history of hypertension.

**Supplementary Information:**

The online version contains supplementary material available at 10.1186/s12883-021-02252-2.

## Introduction

Although stroke severity and age are known to be powerful outcome predictors after acute ischemic stroke (AIS) [1–3], the prognosis of stroke patients is highly variable, and many previous studies have tried to identify outcome predictors in these patients to help guide treatment decisions [1]. Vital signs can help to detect or monitor medical problems, and many vital sign parameters have been associated with functional outcomes after stroke [4–6]. Although the effects of blood pressure (BP) on functional outcomes, mortality, and vascular outcomes after stroke have been well characterized, few studies have investigated the effects of heart rate (HR) [7–12].

In older adults, lower HR variability and higher HR at rest have been associated with poor functional status and an increased risk of a subsequent decline in functional status independently of cardiovascular disease [13]. The Framingham study indicated that resting HR can be used to predict cardiovascular death in the general population [14]. In addition, a higher risk of mortality has been associated with a high resting HR independently of physical leisure activity and fitness and other well-known cardiovascular risk factors [15]. Moreover, in patients with coronary artery disease and hypertension, an elevated HR at rest has been reported to be a predictive factor for overall and cardiovascular mortality independently of other risk factors [16, 17].

Reducing HR was shown to protect mice from cerebral ischemia by reducing oxidative stress and improving endothelial function [18]. Patients with increased tachycardia burden during hospitalization for stroke have also been reported to have poor functional outcomes [19]. In addition, the Prevention Regimen for Effectively Avoiding Second Strokes (PRoFESS) study reported that resting HR was an important prognostic factor for stroke survivors, and Bohm et al. reported that an elevated HR was strongly associated with the prognosis independently of co-variables including high BP [20]. However, another study reported that significant tachycardia and bradycardia could not independently predict the clinical course or outcomes in stroke patients [21].

Although HR variability measured by variations in the beat-to-beat interval has been shown to be an independent predictor of outcomes in patients with AIS [22, 23], few studies have investigated the correlation between visit-to-visit HR variability and stroke outcomes. Therefore, the aim of this study was to investigate the association between initial in-hospital HR and visit-to-visit variations with 3-month functional outcomes after AIS.

## Material and methods

### Subjects and data collection

Patients admitted for AIS at Chiayi Chang Gung Memorial Hospital who arrived within 3 days of symptom onset were consecutively identified between October 2013 and July 2018. Ischemic stroke was defined as the sudden onset of neurologic dysfunction caused by focal cerebral infarction and confirmed by imaging studies [24]. Key demographic and clinical characteristics were collected from the Stroke Registry in Chang Gung Healthcare System (SRICHS), including stroke severity as measured using the National Institute of Health Stroke Scale (NIHSS) at baseline [25]. The NIHSS score was assessed on admission by trained stroke neurologists. The recorded vital sign values for the enrolled subjects during the acute stage were downloaded from the Chang Gung Research Database [26], the largest multi-institutional electronic medical records collection in Taiwan, including systolic BP (SBP), diastolic BP (DBP), and HR. Routine hospital management strategies were based on current stroke guidelines [27]. The mean SBP, DBP, and HR and the coefficient of variation (CV) of HR (HR-CV) were derived from the vital sign values recorded during the initial 3 days after admission. CV was chosen as a measure of variation because it is more independent of the mean than standard deviation.

Estimated glomerular filtration rate (eGFR) was determined using the equation proposed by the Taiwan Society of Nephrology as follows: estimated glomerular filtration rate (mL/min/1.73 m^2^) = 186 × (serum creatinine)^−1.154^ × (age)^−0.203^ × 0.742 (if female). Thrombolytic therapy was defined as the intravenous administration of recombinant tissue-type plasminogen activator or intra-arterial thrombolysis in the acute phase of the stroke.

### Study outcomes

The study outcome was death or major disability (unfavorable functional outcome), defined as a score of 3–6 on the modified Rankin Scale (scores ranging from 0 [no symptoms] to 5 [severe disability] and 6 [death]) at 3 months [28]. Information on functional status was collected via direct or telephone interviews with the patients, family members or caregivers by trained and certified research nurses.

### Statistical analysis

All analyses were performed using SPSS for Windows, version 22.0 (SPSS Inc., Chicago, IL, USA), and a *P* value of < 0.05 was considered to indicate a statistically significant difference. Quantitative variables were summarized as mean (standard deviation) or median (interquartile range), depending on the distribution of the data, and categorical variables were presented as number (percentage). Multivariate logistic regression analysis was used to evaluate the relationship between HR and 3-month functional outcomes. Formal analyses were performed using HR and HR-CV as a continuous and as a categorical variables as well. Odds ratios (ORs) and 95% confidence interval (CIs) were calculated. In addition to crude ORs, adjusted ORs were estimated after adjustments for potential confounding factors in the multivariate logistic regression analysis. C-statistics were calculated to assess the predictive value of the multivariate model using the mean HR or HR-CV for functional outcomes.

## Results

### Subject demographics

**Fig. 1:**
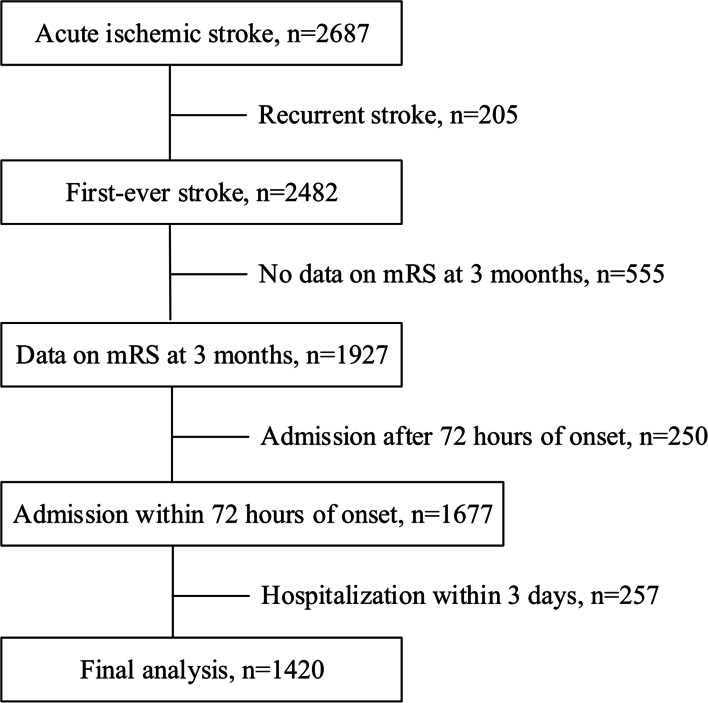
Flow chart of patient selection. Abbreviation: mRS, modified Rankin Scale

Among 2,687 patients who had AIS and were admitted to Chiayi Chang Gung Memorial Hospital in the SRICHS, 2,482 patients were defined as having their first ever ischemic stroke. Of these patients, 555 were lost during the 3-month follow-up period, and 507 who had other specified conditions were excluded (Fig. 1). The remaining 1,420 patients were included in this study (mean age, 69.8 ± 14.3 years; 54.3% males). The clinical characteristics of the included patients are presented in Table 1. Overall, 37.7% of the patients had unfavorable 3-month functional outcomes after AIS.

**Table 1:** Characteristics of the patients

Clinical Background	*N* = 1420
Age, y, mean ± SD	69.8 ± 14.3
Men, n (%)	813 (54.3)
Hypertension, n (%)	1076 (75.8)
Diabetes mellitus, n (%)	616 (43.4)
Dyslipidemia, n (%)	903 (63.6)
Atrial fibrillation, n (%)	287 (20.2)
Coronary artery disease, n (%)	94 (6.6)
Congestive heart failure, n (%)	58 (4.1)
Thrombolytic therapy, n (%)	147 (10.4)
Body mass index, kg/m^2^, mean ± SD	24.6 ± 4.1
Current smoker, n (%)	341 (24.0)
Cholesterol, mmol/L, mean ± SD	4.50 ± 1.12
Triglyceride, mmol/L, mean ± SD	1.38 ± 1.01
eGFR, mL/min per 1.73 m^2^, mean ± SD	62.6 ± 29.1
Mean systolic blood pressure, mmHg, mean ± SD	151.9 ± 21.2
Mean diastolic blood pressure, mmHg, mean ± SD	85.8 ± 12.2
NIHSS on admission, median (IQR)	4 (1–7)
Unfavorable 3-month functional outcome, n (%)	535 (37.7)
Stroke subtypes, n (%)	
Atherothrombotic	228 (16.1)
Cardioembolic	295 (20.8)
Lacunar	534 (37.6)
Other determined pathogenesis	23 (1.6)
Undetermined pathogenesis	340 (23.9)

Data are n (%) for categorical data and mean (standard deviation, SD) or median (interquartile range, IQR) for continuous data, depending on the distribution of the data

*Abbreviations*: *eGFR* estimated glomerular filtration rate, *NIHSS* National Institute of Health Stroke Scale

### Association between the HR and three-month functional outcome in total stroke patients

The demographic and baseline data of the overall cohort stratified by HR (< 70 beats per minute (bpm), ≥ 70 and < 80 bpm, ≥ 80 and < 90 bpm, and ≥ 90 bpm) and HR-CV (< 0.08, ≥ 0.08 and < 0.10, ≥ 0.10 and < 0.12, ≥ 0.12) are shown in Tables 2 and 3, respectively. The effects of the mean initial in-hospital HR and HR-CV levels on the risk of an unfavorable functional outcome are summarized in Table 4. When the NIHSS score was divided into three groups (mild, 0–6; moderate, 7–16; and severe, 17–40), there was a significant increase in the mean HR value across the groups (71.4 ± 11.3 bpm in the mild group, 79.1 ± 13.0 bpm in the moderate group, and 88.9 ± 14.6 bpm in the severe group, *P* for trend < 0.001). There was also a significant increase in HR-CV across the three NIHSS score groups (0.10 ± 0.04 in the mild group, 0.12 ± 0.05 in the moderate group, and 0.13 ± 0.06 in the severe group, *P* for trend < 0.001) (Supplementary Table 1). The multivariate models included age, sex, dyslipidemia, atrial fibrillation, congestive heart failure, smoking status, body mass index, thrombolytic therapy, mean SBP, NIHSS score, and the levels of total cholesterol and eGFR for analysis including mean HR (Supplementary Tables 2 and 3); and age, diabetes mellitus, dyslipidemia, atrial fibrillation, congestive heart failure, thrombolytic therapy, mean SBP, NIHSS score, and the levels of triglycerides and eGFR for analysis including HR-CV (Supplementary Tables 4 and 5).

**Table 2:** Demographic and baseline characteristics of the overall cohort stratified by mean heart rate

	Mean HR categories				
	< 70 bpm	≥ 70 and < 80 bpm	≥ 80 and < 90 bpm	≥ 90 bpm	
Parameter	(*N* = 570)	(*N* = 404)	(*N* = 262)	(*N* = 184)	*P* value
Age (years)	68.7 (13.5)	69.3 (14.8)	70.6 (15.8)	73.4 (12.9)	0.001
Men	347 (60.9)	235 (58.2)	135 (51.5)	96 (52.2)	0.034
Hypertension	424 (74.4)	314 (77.7)	208 (79.4)	130 (70.7)	0.115
Diabetes mellitus	228 (40.0)	177 (43.8)	128 (48.9)	83 (45.1)	0.107
Dyslipidemia	384 (67.4)	263 (65.1)	166 (63.4)	90 (48.9)	< 0.001
Atrial fibrillation	65 (11.4)	63 (15.6)	68 (26.0)	91 (49.5)	< 0.001
Coronary artery disease	33 (5.8)	28 (6.9)	18 (6.9)	15 (8.2)	0.700
Congestive heart failure	14 (2.5)	21 (5.2)	11 (4.2)	12 (6.5)	0.047
Body mass index (kg/m^2^)	24.8 (3.7)	24.4 (4.2)	24.9 (4.3)	23.7 (4.5)	0.008
Current smoker	178 (31.2)	87 (21.5)	45 (17.2)	31 (16.8)	< 0.001
Total cholesterol (mmol/L)	4.60 (1.06)	4.58 (1.10)	4.52 (1.16)	4.22 (1.30)	0.002
Triglyceride (mmol/L)	1.37 (0.96)	1.42 (1.13)	1.40 (0.94)	1.27 (1.00)	0.422
eGFR (mL/min per 1.73 m^2^)	65.5 (28.0)	63.5 (27.8)	60.8 (30.4)	54.6 (31.7)	< 0.001
Thrombolytic therapy	48 (8.4)	38 (9.4)	25 (9.5)	36 (19.6)	< 0.001
Mean SBP (mmHg)	154.4 (21.3)	151.6 (20.5)	151.9 (22.6)	145.1 (19.2)	< 0.001
Mean DBP (mmHg)	85.2 (11.5)	86.3 (11.5)	87.1 (13.3)	84.7 (13.8)	0.093
NIHSS score on admission	2 (1–4)	4 (1–7)	4 (2–9)	12 (5–21)	< 0.001

Data are n (%) for categorical data and mean (standard deviation) or median (interquartile range) for continuous data, depending on the distribution of the data

*Abbreviations*: *HR* heart rate, *SBP* systolic blood pressure, *DBP* diastolic blood pressure, *eGFR* estimated glomerular filtration rate, *NIHSS* National Institute of Health Stroke Scale

**Table 3:** Demographic and baseline characteristics of the overall cohort stratified by the coefficient of variation of heart rate

	HR-CV categories				
	< 0.08	≥ 0.08 and < 0.10	≥ 0.10 and < 0.12	≥ 0.12	
Parameter	*N* = 397	*N* = 305	*N* = 236	*N* = 482	*P* value
Age (years)	68.5 (14.6)	69.3 (14.2)	69.7 (14.0)	71.3 (14.3)	0.035
Men	216 (54.4)	163 (53.4)	139 (58.9)	295 (61.2)	0.091
Hypertension	310 (78.1)	238 (78.0)	170 (72.0)	358 (74.3)	0.222
Diabetes mellitus	205 (51.6)	132 (43.3)	108 (45.8)	171 (35.5)	< 0.001
Dyslipidemia	278 (70.0)	191 (62.6)	161 (68.2)	273 (56.6)	< 0.001
Atrial fibrillation	39 (9.8)	54 (17.7)	55 (23.3)	139 (28.8)	< 0.001
Coronary artery disease	33 (8.3)	21 (6.9)	16 (6.8)	24 (5.0)	0.263
Congestive heart failure	19 (4.8)	8 (2.6)	10 (4.2)	21 (4.4)	0.519
Body mass index (kg/m^2^)	24.7 (4.1)	24.6 (3.7)	24.5 (4.2)	24.5 (4.4)	0.819
Current smoker	103 (25.9)	68 (22.3)	63 (28.0)	103 (21.4)	0.163
Total cholesterol (mmol/L)	4.54 (1.11)	4.59 (1.07)	4.56 (1.05)	4.43 (1.20)	0.164
Triglyceride (mmol/L)	1.52 (1.21)	1.39 (0.86)	1.45 (1.23)	1.21 (0.75)	< 0.001
eGFR (mL/min per 1.73 m^2^)	63.6 (28.7)	64.2 (30.7)	64.7 (32.8)	60.0 (26.3)	0.090
Thrombolytic therapy	18 (4.5)	30 (9.8)	30 (12.7)	69 (14.3)	< 0.001
Mean SBP (mmHg)	153.4 (22.4)	153.6 (21.3)	151.5 (20.9)	150.0 (20.2)	0.050
Mean DBP (mmHg)	86.9 (11.5)	86.1 (12.6)	85.5 (12.8)	84.9 (12.1)	0.086
NIHSS score on admission	3 (1–6)	3 (1–6)	4 (2–9)	4 (1–10)	0.01

Data are n (%) for categorical data and mean (standard deviation) or median (interquartile range) for continuous data, depending on the distribution of the data

*Abbreviations*: *HR-CV* the coefficient of variation of heart rate, *SBP* systolic blood pressure, *DBP* diastolic blood pressure, *eGFR* estimated glomerular filtration rate, *NIHSS* National Institute of Health Stroke Scale

**Table 4:** Association between heart rate and unfavorable 3-month functional outcomes in the overall cohort

HR subgroup	Number of events/at risk, %	Unadjusted			Multivariate-adjusted		
		OR	95% CI	*P* value	OR	95% CI	*P* value
Mean HR							
G1 (< 70 bpm)	119/570 (21)	1.00	Reference	…	1.00	Reference	…
G2 (≥ 70 and < 80 bpm)	153/404 (38)	2.31	1.74 − 3.07	< 0.001	1.81	1.25 − 2.61	0.002
G3 (≥ 80 and < 90 bpm)	129/262 (49)	3.68	2.68 − 5.04	< 0.001	2.52	1.66 − 3.82	< 0.001
G4 (≥ 90 bpm)	134/184 (73)	10.16	6.93 − 14.89	< 0.001	3.88	2.20 − 6.85	< 0.001
*P* for trend				< 0.001			< 0.001
HR-CV							
G1 (< 0.08)	124/397 (31)	1.00	Reference	…	1.00	Reference	…
G2 (> 0.08 and < 0.10)	108/305 (35)	1.21	0.88 − 1.66	0.244	1.32	0.87 − 2.00	0.192
G3 (≥ 0.10 and < 0.12)	94/236 (40)	1.46	1.04 − 2.04	0.028	1.10	0.69 − 1.75	0.700
G4 (≥ 0.12)	209/482 (43)	1.69	1.28 − 2.23	< 0.001	1.47	1.00 − 2.16	0.051
*P* for trend				0.002			0.224

*Abbreviations*: *HR* heart rate, *HR-CV* the coefficient of variation of heart rate, *CI* confidence interval, *OR* odds ratio

Compared with the reference group (mean HR < 70 bpm), the adjusted ORs for unfavorable functional outcomes were 1.81 (95% CI, 1.25–2.61) for a mean HR ≥ 70 and < 80 bpm, 2.52 (95% CI, 1.66 − 3.52) for a mean HR ≥ 80 and < 90 bpm, and 3.88 (95% CI, 2.20–6.85) for a mean HR ≥ 90 bpm. A higher mean HR was significantly and continuously associated with a lower probability of a favorable functional outcome. Likewise, the probability of an unfavorable functional outcome was higher for the patients with a HR-CV ≥ 0.12 compared with those with a HR-CV < 0.08 (unadjusted OR, 1.69; 95% CI, 1.28 − 2.23; *P* < 0.001); however, this association was no longer significant after multiple adjustments for potential confounding factors (*P* = 0.051).

### Association between the HR and three-month functional outcome in stroke patients with history of hypertension

In order to evaluate the association between HR and functional outcomes in the patients with a history of hypertension, we also performed multivariate logistic regression analysis in this group of patients. The demographic and baseline data of the patients with a history of hypertension stratified into four groups by mean HR and HR-CV are given in Supplementary Tables 6 and 7, respectively. Table 5 shows the relationships between the initial in-hospital HR and 3-month functional outcomes in the patients with a history of hypertension. The multivariate models included age, body mass index, dyslipidemia, atrial fibrillation, congestive heart failure, smoking status, eGFR, total cholesterol, thrombolytic therapy, mean SBP, and baseline NIHSS score for analysis including mean HR; and age, diabetes, dyslipidemia, atrial fibrillation, congestive heart failure, triglycerides, thrombolytic therapy, mean SBP, mean DBP, and baseline NIHSS score for analysis including HR-CV.

**Table 5:** Association between heart rate and unfavorable 3-month functional outcomes in the patients with a history of hypertension

HR subgroup	Number of events/at risk, %	Unadjusted			Multivariate-adjusted		
		OR	95% CI	*P* value	OR	95% CI	*P* value
Mean HR							
G1 (< 70 bpm)	97/424 (23)	1.00	Reference	…	1.00	Reference	…
G2 (≥ 70 and < 80 bpm)	122/314 (39)	2.14	1.55 − 2.95	< 0.001	1.80	1.18 − 2.73	0.006
G3 (≥ 80 and < 90 bpm)	102/208 (49)	3.24	2.28 − 4.62	< 0.001	2.35	1.47 − 3.76	< 0.001
G4 (≥ 90 bpm)	95/130 (73)	9.15	5.84 − 14.34	< 0.001	2.96	1.51 − 5.81	0.002
*P* for trend				< 0.001			< 0.001
HR-CV							
G1 (< 0.08)	99/310 (32)	1.00	Reference	…	1.00	Reference	…
G2 (> 0.08 and < 0.10)	89/238 (37)	1.27	0.89 − 1.82	0.182	1.59	0.99 − 2.54	0.053
G3 (≥ 0.10 and < 0.12)	69/170 (41)	1.46	0.99 − 2.15	0.058	1.40	0.81 − 2.40	0.227
G4 (≥ 0.12)	159/358 (44)	1.70	1.24 − 2.34	0.001	1.73	1.11 − 2.70	0.015
*P* for trend				0.010			0.086

*Abbreviations*: *HR* heart rate, *HR-CV* the coefficient of variation of heart rate, *CI* confidence interval, *OR* odds ratio

For patients with a history of hypertension, a higher mean HR level was also associated with an increased risk of an unfavorable functional outcome as in the overall study cohort. In addition, the probability of an unfavorable functional outcome was higher in the patients with a HR-CV ≥ 0.12 compared with those with a HR-CV < 0.08, even after multiple adjustments for potential confounding factors (adjusted OR, 1.73; 95% CI, 1.11 − 2.70; *P* = 0.015) (Table 5).

We calculated C-statistics in which mean HR and HR-CV were separately included in the multivariate models for the overall cohort and the patients with a history of hypertension, respectively. The C-statistics for an unfavorable functional outcome were 0.883 for analysis including the mean HR in the overall cohort and 0.879 for analysis including HR-CV in the patients with a history of hypertension.

## Discussion

In this study, we showed that an increased mean initial in-hospital HR was associated with an unfavorable 3-month functional outcome in patients with AIS. In addition, for the AIS patients with a history of hypertension, compared with those with a HR-CV < 0.08, patients with a HR-CV ≥ 0.12 also had a higher probability of an unfavorable functional outcome.

Previous studies have reported inconsistent results with regards to the relationship between post-stroke HR and functional outcomes. Ritter et al. did not find associations between significant tachycardia and bradycardia and clinical outcomes [21]. However, other studies have suggested that an increased HR was unfavorable with regards to outcomes [4, 19, 20, 29–31]. In the PRoFESS study, there was a better 3-month functional outcome after recurrent stroke in patients with a lower HR [20]. In the present study, we also found that the probability of unfavorable functional outcomes increased progressively as the level of mean initial in-hospital HR increased.

The mechanisms underlying the association between post-stroke HR and clinical outcomes are currently unknown. Bohm et al. reported that a high resting HR was associated with the progression of atherosclerosis through a negative effect on the endothelium [32]. In addition, Custodis et al. demonstrated that reducing HR with ivabradine, an I(f) current inhibitor, could restore endothelial function, reduce oxidative stress, and protect against focal brain ischemia by a markedly reducing the size of cerebral lesions in mice [18]. A high HR may also reflect sympathetic nervous overactivity, which has been associated with inflammatory responses and an elevated BP at night, both of which are well-known predictors of mortality in stroke patients [33–35]. We also found that the mean initial in-hospital HR was associated with stroke severity in the present study (Supplementary Table 1).

Although the effects of HR variability measured by the variation in beat-to-beat interval and visit-to-visit BP variability on functional and vascular outcomes after stroke have been well characterized [22, 23, 36–39], few studies have investigated visit-to-visit HR variability. In the Ohasama study, day-by-day HR variability was associated with cardiovascular and cardiac mortality, but not stroke mortality [40]. Yang et al. reported that increased resting HR variability combined with an increase in long-term visit-to-visit SBP variability or vice versa may increase the risk of all-cause mortality in the general population [41]. In this study, a high HR-CV was not significantly associated with functional outcomes in the overall cohort, however, for patients with a history of hypertension, the risk of an unfavorable functional outcome was higher in the patients with a HR-CV ≥ 0.12 compared with those with a HR-CV < 0.08. Although the mechanisms for outcome prediction of visit-to-visit HR variation remains unknown, we speculate that the effect of visit-to-visit HR variation on functional outcome is more pronounced in patients with a history of hypertension.

There are several limitations to this study. We determined the mean HR and HR-CV levels by using initial 3-day measurements and did not record the values at the same interval during hospitalization. In addition, the number of vital sign measurements was different for each patient, which may have resulted in both underestimation and overestimation of the association between the post-stroke HR levels and the study outcome. Another limitation is the lack of follow-up of all patients with AIS. The reason for this is a change in address and contact numbers of many of the patients admitted to our hospital, since most of them were elderly and their caregivers usually lived far from the hospital which limited their recruitment for the follow-up study.

## Conclusion

Our data showed that a higher initial in-hospital HR was associated with an unfavorable functional outcome at 3 months in patients with AIS. However, these findings do not encourage the use of rate-slowing agents for patients with AIS at this stage. Further studies are required to elucidate the causality between HR and functional outcomes after AIS. Nevertheless, our findings suggest the potential role of HR and its modulation in future cardiovascular guidelines.

## Supplementary Information

**Additional file 1.**

## Data Availability

The data supporting the findings of the article is available in the Chang Gung Research Databank at Chang Gung Memorial Hospital, Chiayi Branch. These data can be available after obtaining approval from our local IRB.

## Abbreviations

AIS: Acute ischemic stroke
BP: Blood pressure
HR: Heart rate
PRoFESS: Prevention Regimen for Effectively Avoiding Second Strokes
NIHSS: National Institute of Health Stroke Scale
SRICHS: Stroke Registry in Chang Gung Healthcare System
SBP: Systolic blood pressure
DBP: Diastolic blood pressure
HR-CV: Coefficient of variation of heart rate
eGFR: Estimated glomerular filtration rate
OR: Odds ratio
CI: Confidence interval
bpm: Beats per minute

## References

[1] Veerbeek JM, Kwakkel G, van Wegen EE, Ket JC, Heymans MW (2011). Early prediction of outcome of activities of daily living after stroke: a systematic review. Stroke.

[2] Rost NS, Bottle A, Lee JM, Randall M, Middleton S, Shaw L, Thijs V, Rinkel GJ, Hemmen TM, Global Comparators Stroke Gc (2016). Stroke severity is a crucial predictor of outcome: an international prospective validation study. J Am Heart Assoc.

[3] Wouters A, Nysten C, Thijs V, Lemmens R (2018). Prediction of outcome in patients with acute ischemic stroke based on initial severity and improvement in the first 24 h. Front Neurol.

[4] Shah B, Bartaula B, Adhikari J, Neupane HS, Shah BP, Poudel G (2017). Predictors of in-hospital mortality of acute ischemic stroke in adult population. J Neurosci Rural Pract.

[5] Geurts M, Scheijmans FE, van Seeters T, Biessels GJ, Kappelle LJ, Velthuis BK, van der Worp HB, investigators D (2016). Temporal profile of body temperature in acute ischemic stroke: relation to infarct size and outcome. BMC Neurol.

[6] Graff B, Gasecki D, Rojek A, Boutouyrie P, Nyka W, Laurent S, Narkiewicz K (2013). Heart rate variability and functional outcome in ischemic stroke: a multiparameter approach. J Hypertens.

[7] Ovbiagele B, Diener HC, Yusuf S, Martin RH, Cotton D, Vinisko R, Donnan GA, Bath PM, Investigators P (2011). Level of systolic blood pressure within the normal range and risk of recurrent stroke. JAMA.

[8] Bager JE, Hjalmarsson C, Manhem K, Andersson B (2018). Acute blood pressure levels and long-term outcome in ischemic stroke. Brain Behav.

[9] Stead LG, Gilmore RM, Vedula KC, Weaver AL, Decker WW, Brown RD (2006). Impact of acute blood pressure variability on ischemic stroke outcome. Neurology.

[10] Hong KS (2017). Blood pressure management for stroke prevention and in acute stroke. J Stroke.

[11] Lattanzi S, Brigo F, Silvestrini M (2019). Blood pressure and stroke: from incidence to outcome. J Clin Hypertens (Greenwich).

[12] Ishitsuka K, Kamouchi M, Hata J, Fukuda K, Matsuo R, Kuroda J, Ago T, Kuwashiro T, Sugimori H, Nakane H (2014). High blood pressure after acute ischemic stroke is associated with poor clinical outcomes: Fukuoka Stroke Registry. Hypertension.

[13] Ogliari G, Mahinrad S, Stott DJ, Jukema JW, Mooijaart SP, Macfarlane PW, Clark EN, Kearney PM, Westendorp RGJ, de Craen AJM (2015). Resting heart rate, heart rate variability and functional decline in old age. CMAJ.

[14] Kannel WB, Kannel C, Paffenbarger RS, Cupples LA (1987). Heart rate and cardiovascular mortality: the Framingham Study. Am Heart J.

[15] Jensen MT, Suadicani P, Hein HO, Gyntelberg F (2013). Elevated resting heart rate, physical fitness and all-cause mortality: a 16-year follow-up in the Copenhagen Male Study. Heart.

[16] Diaz A, Bourassa MG, Guertin MC, Tardif JC (2005). Long-term prognostic value of resting heart rate in patients with suspected or proven coronary artery disease. Eur Heart J.

[17] Kolloch R, Legler UF, Champion A, Cooper-Dehoff RM, Handberg E, Zhou Q, Pepine CJ (2008). Impact of resting heart rate on outcomes in hypertensive patients with coronary artery disease: findings from the INternational VErapamil-SR/trandolapril STudy (INVEST). Eur Heart J.

[18] Custodis F, Gertz K, Balkaya M, Prinz V, Mathar I, Stamm C, Kronenberg G, Kazakov A, Freichel M, Bohm M (2011). Heart rate contributes to the vascular effects of chronic mental stress: effects on endothelial function and ischemic brain injury in mice. Stroke.

[19] Jeong HG, Ko SB, Kim CK, Kim Y, Jung S, Kim TJ, Yoon BW (2016). Tachycardia burden in stroke unit is associated with functional outcome after ischemic stroke. Int J Stroke.

[20] Bohm M, Cotton D, Foster L, Custodis F, Laufs U, Sacco R, Bath PM, Yusuf S, Diener HC (2012). Impact of resting heart rate on mortality, disability and cognitive decline in patients after ischaemic stroke. Eur Heart J.

[21] Ritter MA, Rohde A, Heuschmann PU, Dziewas R, Stypmann J, Nabavi DG, Ringelstein BE (2011). Heart rate monitoring on the stroke unit. What does heart beat tell about prognosis? An observational study. BMC Neurol.

[22] Gujjar AR, Sathyaprabha TN, Nagaraja D, Thennarasu K, Pradhan N (2004). Heart rate variability and outcome in acute severe stroke: role of power spectral analysis. Neurocrit Care.

[23] Lees T, Shad-Kaneez F, Simpson AM, Nassif NT, Lin Y, Lal S (2018). Heart rate variability as a biomarker for predicting stroke post-stroke complications and functionality. Biomark Insights.

[24] Sacco RL, Kasner SE, Broderick JP, Caplan LR, Connors JJ, Culebras A, Elkind MS, George MG, Hamdan AD, Higashida RT (2013). An updated definition of stroke for the 21st century: a statement for healthcare professionals from the American Heart Association/American Stroke Association. Stroke.

[25] Lee TH, Chang CH, Chang YJ, Chang KC, Chung J, Chang Gung Medical System Stroke Registry G (2011). Establishment of electronic chart-based stroke registry system in a medical system in Taiwan. J Formos Med Assoc.

[26] Tsai MS, Lin MH, Lee CP, Yang YH, Chen WC, Chang GH, Tsai YT, Chen PC, Tsai YH (2017). Chang Gung Research Database: a multi-institutional database consisting of original medical records. Biomed J.

[27] Jauch EC, Saver JL, Adams HP, Bruno A, Connors JJ, Demaerschalk BM, Khatri P, McMullan PW, Qureshi AI, Rosenfield K (2013). Guidelines for the early management of patients with acute ischemic stroke: a guideline for healthcare professionals from the American Heart Association/American Stroke Association. Stroke.

[28] van Swieten JC, Koudstaal PJ, Visser MC, Schouten HJ, van Gijn J (1988). Interobserver agreement for the assessment of handicap in stroke patients. Stroke.

[29] Erdur H, Scheitz JF, Grittner U, Laufs U, Endres M, Nolte CH (2014). Heart rate on admission independently predicts in-hospital mortality in acute ischemic stroke patients. Int J Cardiol.

[30] Sandset EC, Berge E, Kjeldsen SE, Julius S, Holzhauer B, Krarup LH, Hua TA (2014). Heart rate as a predictor of stroke in high-risk, hypertensive patients with previous stroke or transient ischemic attack. J Stroke Cerebrovasc Dis.

[31] Nakicevic A, Alajbegovic S, Alajbegovic L (2017). Tachycardia as a negative prognostic factor for stroke outcome. Mater Sociomed.

[32] Bohm M, Reil JC (2007). Perspectives of I(f) inhibition by ivabradine in cardiology. Drugs.

[33] Sander D, Winbeck K, Klingelhofer J, Etgen T, Conrad B (2001). Prognostic relevance of pathological sympathetic activation after acute thromboembolic stroke. Neurology.

[34] Winklewski PJ, Radkowski M, Demkow U (2014). Cross-talk between the inflammatory response, sympathetic activation and pulmonary infection in the ischemic stroke. J Neuroinflammation.

[35] den Hertog HM, van Rossum JA, van der Worp HB, van Gemert HM, de Jonge R, Koudstaal PJ, Dippel DW, investigators P (2009). C-reactive protein in the very early phase of acute ischemic stroke: association with poor outcome and death. J Neurol.

[36] Yu JM, Kong QY, Schoenhagen P, Shen T, He YS, Wang JW, Zhao YP, Shi DN, Zhong BL (2014). The prognostic value of long-term visit-to-visit blood pressure variability on stroke in real-world practice: a dynamic cohort study in a large representative sample of Chinese hypertensive population. Int J Cardiol.

[37] de Havenon A, Fino NF, Johnson B, Wong KH, Majersik JJ, Tirschwell D, Rost N (2019). blood pressure variability and cardiovascular outcomes in patients with prior stroke: a secondary analysis of PRoFESS. Stroke.

[38] Dai H, Lu Y, Song L, Tang X, Li Y, Chen R, Luo A, Yuan H, Wu S (2017). Visit-to-visit variability of blood pressure and risk of stroke: results of the Kailuan Cohort Study. Sci Rep.

[39] Fyfe-Johnson AL, Muller CJ, Alonso A, Folsom AR, Gottesman RF, Rosamond WD, Whitsel EA, Agarwal SK, MacLehose RF (2016). Heart rate variability and incident stroke: the atherosclerosis risk in communities study. Stroke.

[40] Kikuya M, Ohkubo T, Metoki H, Asayama K, Hara A, Obara T, Inoue R, Hoshi H, Hashimoto J, Totsune K (2008). Day-by-day variability of blood pressure and heart rate at home as a novel predictor of prognosis: the Ohasama study. Hypertension.

[41] Yang X, Hidru TH, Wang B, Han X, Li H, Wu S, Xia Y (2019). The link between elevated long-term resting heart rate and SBP variability for all-cause mortality. J Hypertens.

