# Supplementary material for: Initial in-hospital heart rate is associated with three-month functional outcomes after acute ischemic stroke
Source: BMC Neurol. 2021 Jun 11;21:222. doi: 10.1186/s12883-021-02252-2 (PMC8194208; doi:10.1186/s12883-021-02252-2)
Supplement: Supplementary file 1 — Additional file 1. [file 12883_2021_2252_MOESM1_ESM.docx]

**Initial in-hospital heart rate is associated with three-month functional outcomes after acute ischemic stroke**

Ya-Wen Kuo, PhD^1^, Meng Lee, MD^2^, Yen-Chu Huang, MD^2^, Jiann-Der Lee, MD, PhD^2^

^1^Department of Nursing, Chang Gung University of Science and Technology, Chiayi Campus, Chiayi, Taiwan

^2^Department of Neurology, Chang Gung Memorial Hospital, Chiayi, and College of Medicine, Chang Gung University, Taoyuan, Taiwan

**Supplementary Table 1. Characteristics of the mean heart rate and coefficient of variation of heart rate according to NIHSS score subgroups**

|  | NIHSS score  0-6 | NIHSS score  7-16 | NIHSS score  17-40 | *P* for trend |
| --- | --- | --- | --- | --- |
| Mean HR, beats per minute | 71.4 (11.3) | 79.1 (13.0) | 88.9 (14.6) | < 0.001 |
| HR-CV | 0.10 (0.04) | 0.12 (0.05) | 0.13 (0.06) | < 0.001 |

Continuous variables are presented as the mean (standard deviation). *P* for trend was calculated by analysis of variance.

Abbreviations: NIHSS, National Institute of Health Stroke Scale; HR, heart rate; HR-CV, Coefficient of Variation of heart rate.

**Supplementary Table 2. Multivariable logistic regression analysis including mean heart rate as a continuous variable for 3-month functional outcome in the overall cohort**

|  | Poor functional outcome |  |
| --- | --- | --- |
|  | OR (95% CI) | *P* value |
| Mean HR | 1.04 (1.03–1.06) | < 0.001 |
| Age | 1.07 (1.05–1.09) | < 0.001 |
| Male gender | 0.74 (0.52–1.05) | 0.094 |
| Dyslipidemia | 0.98 (0.69–1.39) | 0.923 |
| Atrial fibrillation | 0.50 (0.32–0.79) | 0.003 |
| Congestive heart failure | 1.22 (0.55–2.73) | 0.629 |
| Current smoker | 0.88 (0.57–1.37) | 0.581 |
| Body mass index | 0.97 (0.93–1.01) | 0.131 |
| Thrombolytic therapy | 0.88 (0.52–1.49) | 0.630 |
| Mean SBP | 1.00 (0.99–1.01) | 0.741 |
| NIHSS score | 1.29 (1.24–1.35) | < 0.001 |
| Cholesterol | 1.00 (1.00–1.01) | 0.732 |
| eGFR | 1.00 (0.99–1.00) | 0.421 |

Abbreviations: OR, odds ratio; CI, confidence interval; HR, heart rate; SBP, systolic blood pressure; eGFR, estimated glomerular filtration rate; NIHSS, National Institute of Health Stroke Scale.

|  | Poor functional outcome |  |
| --- | --- | --- |
|  | OR (95% CI) | *P* value |
| Mean HR subgroup |  |  |
| < 70 bpm | Reference |  |
| ≥ 70, < 80 bpm | 1.81 (1.25–2.61) | 0.002 |
| ≥ 80, < 90 bpm | 2.52 (1.66–3.82) | < 0.001 |
| ≥ 90 bpm | 3.88 (2.20–6.85) | < 0.001 |
| Age | 1.07 (1.05–1.09) | < 0.001 |
| Male gender | 0.74 (0.52–1.04) | 0.085 |
| Dyslipidemia | 1.00 (0.70–1.41) | 0.981 |
| Atrial fibrillation | 0.53 (0.34–0.83) | 0.005 |
| Congestive heart failure | 1.27 (0.57–2.82) | 0.557 |
| Current smoker | 0.90 (0.58–1.40) | 0.642 |
| Body mass index | 0.97 (0.93–1.01) | 0.133 |
| Thrombolytic therapy | 0.87 (0.51–1.47) | 0.606 |
| Mean SBP | 1.00 (0.99–1.01) | 0.753 |
| NIHSS score | 1.29 (1.24–1.35) | < 0.001 |
| Cholesterol | 1.00 (1.00–1.01) | 0.689 |
| eGFR | 1.00 (0.99–1.00) | 0.476 |

**Supplementary Table 3. Multivariable logistic regression analysis including mean heart rate as a categorical variable for 3-month functional outcome in the overall cohort**

Abbreviations: OR, odds ratio; CI, confidence interval; HR, heart rate; SBP, systolic blood pressure; eGFR, estimated glomerular filtration rate; NIHSS, National Institute of Health Stroke Scale.

**Supplementary Table 4. Multivariable logistic regression analysis including the coefficient of variation of heart rate as a continuous variable for 3-month functional outcome in the overall cohort**

|  | Poor functional outcome |  |
| --- | --- | --- |
|  | OR (95% CI) | *P* value |
| HR-CV (per 1 SD) | 1.11 (0.94–1.30) | 0.222 |
| Age | 1.07 (1.06–1.09) | < 0.001 |
| Diabetes mellitus | 1.50 (1.10–2.05) | 0.010 |
| Dyslipidemia | 1.02 (0.74–1.40) | 0.896 |
| Atrial fibrillation | 0.68 (0.45–1.05) | 0.079 |
| Congestive heart failure | 1.22 (0.56–2.65) | 0.619 |
| Thrombolytic therapy | 0.82 (0.49–1.37) | 0.443 |
| Mean SBP | 1.00 (0.99–1.01) | 0.907 |
| NIHSS score | 1.33 (1.28–1.39) | < 0.001 |
| Triglyceride | 1.00 (1.00–1.00) | 0.843 |
| eGFR | 1.00 (0.99–1.00) | 0.384 |

Abbreviations: OR, odds ratio; CI, confidence interval; HR-CV, the coefficient of variation of heart rate; SD, standard deviation; HR, heart rate; SBP, systolic blood pressure; eGFR, estimated glomerular filtration rate; NIHSS, National Institute of Health Stroke Scale.

**Supplementary Table 5. Multivariable logistic regression analysis including the coefficient of variation of heart rate as a categorical variable for 3-month functional outcome in the overall cohort**

|  | Poor functional outcome |  |
| --- | --- | --- |
|  | OR (95% CI) | *P* value |
| HR-CV subgroup |  |  |
| < 0.08 | Reference |  |
| ≥ 0.08, <0.10 | 1.32 (0.87–2.00) | 0.192 |
| ≥ 0.10, <0.12 | 1.10 (0.69–1.75) | 0.698 |
| ≥ 0.12 | 1.47 (1.00–2.16) | 0.051 |
| Age | 1.07 (1.06–1.09) | < 0.001 |
| Diabetes mellitus | 1.52 (1.12–2.08) | 0.008 |
| Dyslipidemia | 1.03 (0.75–1.41) | 0.879 |
| Atrial fibrillation | 0.68 (0.44–1.03) | 0.070 |
| Congestive heart failure | 1.23 (0.56–2.70) | 0.600 |
| Thrombolytic therapy | 0.80 (0.48–1.35) | 0.404 |
| Mean SBP | 1.00 (0.99–1.01) | 0.937 |
| NIHSS score | 1.34 (1.28–1.39) | < 0.001 |
| Triglyceride | 1.00 (1.00–1.00) | 0.785 |
| eGFR | 1.00 (0.99–1.00) | 0.376 |

Abbreviations: OR, odds ratio; CI, confidence interval; HR-CV, the coefficient of variation of heart rate; SD, standard deviation; HR, heart rate; SBP, systolic blood pressure; eGFR, estimated glomerular filtration rate; NIHSS, National Institute of Health Stroke Scale.

**Supplementary Table 6. Demographic and baseline characteristics of the patients with a history of hypertension stratified by mean heart rate**

|  | Mean HR categories |  |  |  |  |
| --- | --- | --- | --- | --- | --- |
|  | < 70 bpm | ≥ 70 and < 80 bpm | ≥ 80 and < 90 bpm | ≥ 90 bpm |  |
| Parameter | (N = 424) | (N = 314) | (N = 208) | (N = 130) | *P* value |
| Age (years) | 70.0 (12.8) | 70.4 (13.7) | 70.7 (14.1) | 74.2 (11.7) | 0.013 |
| Men | 249 (58.7) | 181 (57.6) | 103 (49.5) | 65 (50.0) | 0.073 |
| Diabetes mellitus | 186 (43.9) | 145 (46.2) | 108 (51.9) | 67 (51.5) | 0.183 |
| Dyslipidemia | 294 (69.3) | 212 (67.5) | 140 (67.3) | 68 (52.3) | 0.004 |
| Atrial fibrillation | 43 (10.1) | 50 (15.9) | 49 (23.6) | 67 (51.5) | < 0.001 |
| Coronary artery disease | 27 (6.4) | 24 (7.6) | 13 (6.3) | 13 (10.0) | 0.503 |
| Congestive heart failure | 12 (2.8) | 17 (5.4) | 9 (4.3) | 10 (7.7) | 0.089 |
| Body mass index (kg/m²) | 24.8 (3.7) | 24.4 (4.2) | 24.9 (4.3) | 23.7 (4.5) | 0.008 |
| Current smoker | 178 (31.2) | 87 (21.5) | 45 (17.2) | 31 (16.8) | < 0.001 |
| Total cholesterol (mmol/L) | 4.54 (1.04) | 4.63 (1.12) | 4.61 (1.142) | 4.19 (1.28) | 0.001 |
| Triglyceride (mmol/L) | 1.35 (0.84) | 1.38 (0.80) | 1.48 (0.99) | 1.30 (1.12) | 0.269 |
| eGFR (mL/min per 1.73 m^2^) | 62.8 (26.3) | 62.1 (27.9) | 61.9 (30.4) | 51.1 (26.6) | < 0.001 |
| Thrombolytic therapy | 48 (8.4) | 38 (9.4) | 25 (9.5) | 36 (19.6) | < 0.001 |
| Mean SBP (mmHg) | 159.3 (20.3) | 155.1 (20.2) | 156.0 (21.7) | 148.9 (19.1) | < 0.001 |
| Mean DBP (mmHg) | 86.9 (11.4) | 87.8 (11.7) | 88.9 (12.8) | 86.6 (14.0) | 0.181 |
| NIHSS score on admission | 2 (1-4) | 4 (1-7) | 4 (2-9) | 13 (6-22) | < 0.001 |

Data are n (%) for categorical data and mean (standard deviation) or median (interquartile range) for continuous data, depending on the distribution of the data.

Abbreviations: HR, heart rate; SBP, systolic blood pressure; DBP, diastolic blood pressure; eGFR, estimated glomerular filtration rate; NIHSS, National Institute of Health Stroke Scale.

**Supplementary Table 7. Demographic and baseline characteristics of the patients with a history of hypertension stratified by coefficient of variation of heart rate**

|  | HR-CV categories |  |  |  |  |
| --- | --- | --- | --- | --- | --- |
|  | < 0.08 | ≥ 0.08 and < 0.10 | ≥ 0.10 and < 0.12 | ≥ 0.12 |  |
| Parameter | N = 310 | N = 238 | N = 170 | N = 358 | *P* value |
| Age (years) | 69.7 (14.1) | 70.4 (12.7) | 70.3 (13.0) | 72.1 (13.0) | 0.104 |
| Men | 167 (53.9) | 119 (50.0) | 94 (55.3) | 218 (60.9) | 0.058 |
| Diabetes mellitus | 166 (53.5) | 109 (45.8) | 87 (51.2) | 144 (40.2) | 0.004 |
| Dyslipidemia | 221 (71.3) | 157 (66.0) | 120 (70.6) | 216 (60.3) | 0.014 |
| Atrial fibrillation | 31 (10.0) | 40 (16.8) | 37 (21.8) | 101 (28.2) | < 0.001 |
| Coronary artery disease | 31 (10.0) | 16 (6.7) | 10 (5.9) | 20 (5.6) | 0.134 |
| Congestive heart failure | 17 (5.5) | 7 (2.9) | 5 (2.9) | 19 (5.3) | 0.311 |
| Body mass index (kg/m²) | 24.7 (4.1) | 24.6 (3.7) | 24.5 (4.2) | 24.5 (4.4) | 0.819 |
| Current smoker | 103 (25.9) | 68 (22.3) | 63 (28.0) | 103 (21.4) | 0.163 |
| Total cholesterol (mmol/L) | 4.54 (1.10) | 4.61 (1.08) | 4.61 (1.04) | 4.46 (1.21) | 0.322 |
| Triglyceride (mmol/L) | 1.49 (1.00) | 1.42 (0.91) | 1.46 (1.10) | 1.22 (0.63) | 0.001 |
| eGFR (mL/min per 1.73 m^2^) | 60.6 (27.6) | 62.4 (26.6) | 64.2 (33.0) | 59.0 (26.1) | 0.194 |
| Thrombolytic therapy | 13 (4.2) | 24 (10.1) | 22 (12.9) | 53 (14.8) | < 0.001 |
| Mean SBP (mmHg) | 157.5 (21.9) | 157.7 (20.4) | 156.5 (19.9) | 153.8 (19.8) | 0.059 |
| Mean DBP (mmHg) | 88.6 (11.3) | 87.8 (12.6) | 88.0 (12.6) | 86.1 (12.2) | 0.057 |
| NIHSS score on admission | 3 (1-6) | 3 (1-6) | 4 (2-8) | 4 (1-10) | 0.01 |

Data are n (%) for categorical data and mean (standard deviation) or median (interquartile range) for continuous data, depending on the distribution of the data.

Abbreviations: HR-CV, the coefficient of variation of heart rate; SBP, systolic blood pressure; DBP, diastolic blood pressure; eGFR, estimated glomerular filtration rate; NIHSS, National Institute of Health Stroke Scale.
